# Mometasone Furoate Alleviates PM2.5‐Induced Pyroptosis of Nasal Mucosa Cells via Blocking JAK2/STAT3/AIM2 Inflammasome

**DOI:** 10.1002/iid3.70476

**Published:** 2026-07-14

**Authors:** Bin Shi, Zhiqi Liu, Ge Le

**Affiliations:** ^1^ Department of Otolaryngology Hubei Maternal and Child Health Hospital Wuhan Hubei China; ^2^ Department of Child Healthcare Hubei Maternal and Child Health Hospital Wuhan Hubei China

**Keywords:** AIM2 inflammasome, autophagy, chronic rhinitis, mometasone furoate, pyroptosis

## Abstract

**Objective:**

Mometasone furoate (MF) is a widely used intranasal corticosteroid for the management of allergic and non‐allergic rhinitis. Recent evidence suggests that its therapeutic efficacy may involve mechanisms beyond traditional anti‐inflammatory actions, including modulation of death in nasal mucosa cells. This study aimed to investigate the therapeutic effects of MF on chronic rhinitis, elucidates the underlying molecular mechanisms, and explores its clinical implications in rhinitis management.

**Methods:**

Enzyme‐linked immunosorbent assay was used to detect cytokine release. Reverse transcription quantitative PCR was applied for detecting mRNA expression. Immunofluorescence was used detect LC3 expression. Protein express was detected using Western blot. Lactate dehydrogenase leakage assay was used to detect cytotoxicity. Terminal deoxynucleotidyl transferase dUTP nick‐end labeling assay was used to detect death of nasal mucosa cells (NMCs). Luciferase and chromatin immunoprecipitation assays were used to verify the interaction between signal transducer and activator of transcription 3 (STAT3) and absent in melanoma 2 (AIM2).

**Results:**

MF significantly suppressed inflammatory response induced by PM2.5. MF suppressed PM2.5‐induced cytotoxicity and pyroptosis of NMCs. Moreover, MF promotes the activation of autophagy. Mechanically, MF inhibited PM2.5‐induced activation of Janus kinase 2/STAT3 signaling. STAT3 transcriptionally upregulated AIM2. The activation of AIM2 inflammasome attenuated the effects of MF and promoted the inflammation and pyroptosis of NMCs as well as suppressed the activation of autophagy.

**Conclusion:**

In summary, MF protects against chronic rhinitis via suppressing AIM2‐dependent pyroptosis of NMCs. Therefore, MF can be a therapeutic strategy for chronic rhinitis.

## 1 Introduction

Chronic rhinitis is a common inflammatory disorder characterized by persistent nasal symptoms, including nasal congestion, rhinorrhea, and sneezing [1]. Chronic rhinitis has become a major chronic inflammatory respiratory disease and is considered one of the most difficult diseases to treat globally [2]. It is often associated with chronic inflammation of the nasal mucosa, leading to tissue remodeling and impaired mucociliary function [3]. Recent studies have highlighted the role of pyroptosis, a form of inflammatory cell death, in the pathogenesis of chronic rhinitis and related conditions such as chronic rhinosinusitis with nasal polyps (CRSwNP) [4]. Therefore, to explore the underlying mechanisms is of vital essence.

Pyroptosis is mediated by the inflammasome complex, which includes AIM2, NLRP3, and other components [5]. Absent in melanoma 2 (AIM2) is a cytosolic sensor that detects dsDNA and activates caspase‐1, leading to the maturation and release of pro‐inflammatory cytokines such as interleukin 1β (IL‐1β) and IL‐18 [6, 7]. Recent studies have demonstrated that the activation of AIM2 inflammasome contribute to the progression of rhinitis. For instance, the activation of AIM2 inflammasome promotes the death of nasal mucosa cells [8]. The activation of AIM2 inflammasome mediates the infiltration of pro‐inflammatory immune cells and the progression of chronic rhinosinusitis [9].

Mometasone furoate (MF), a second‐generation intranasal corticosteroids, has demonstrated significant efficacy in reducing nasal symptoms and improving quality of life in patients with both allergic rhinitis and non‐allergic rhinitis [10, 11]. Recently, increasing reports focus on the anti‐inflammatory properties in the treatment of rhinitis. For instance, 1‐week treatment of MF effectively inhibits the inflammatory response in chronic rhinitis. For instance, MF treatment alleviates eosinophilia‐induced chronic rhinosinusitis [12]. Indolfi et al. [13] demonstrate that MF improves the severity of pediatric rhinoconjunctivitis. However, the precise mechanisms underlying its therapeutic effects are not fully understood.

This study explored the effects of MF on chronic rhinitis and the underlying mechanisms. Human nasal mucosa cells (NMCs) were exposed to PM_2.5_ to induce chronic rhinitis model. We hypothesized that MF protects against PM2.5‐indued chronic rhinitis via blocking inflammatory Janus kinase 2 (JAK2)/signal transducer and activator of transcription 3 (STAT3)/AIM2 signaling, providing a foundation for developing targeted therapeutic strategies for chronic rhinitis.

## 2 Materials and Methods

### 2.1 Cell Culture and Treatment

Human NMCs were isolated from patients with chronic rhinitis and healthy controls. Cells were cultured in Dulbecco's modified Eagle's medium/F12 medium supplemented with 10% fetal bovine serum (FBS) and 1% penicillin‐streptomycin. For experimental treatments, NMCs were stimulated with PM2.5 (50 µg/mL). NMCs were treated with MF (10^−5^M) and/or 3‐Methyladenine (3‐MA) (10 μmol/L).

### 2.2 Cell Transfection

The overexpression plasmids of AIM2 and its vector were provided by GenePharm (Shanghai, China). Cells were transfected using Lipofectamine 2000 (Thermo Fisher Scientific, USA) for 48 h. Then cells were used in the following experiments.

### 2.3 Reverse Transcription Quantitative PCR (RT‐qPCR)

Total RNA was extracted using the RNeasy Mini Kit (Qiagen). cDNA was synthesized using the iScript cDNA Synthesis Kit (Bio‐Rad). Real‐time PCR was performed using SYBR Green Master Mix (Applied Biosystems) to quantify the expression of AIM2, caspase‐1, IL‐1β, and IL‐18. Relative expression levels were normalized to glyceraldehyde‐3‐phosphate dehydrogenase.

### 2.4 Western Blot Analysis

Protein lysates were prepared using radioimmunoprecipitation assay buffer supplemented with protease inhibitors. Equal amounts of protein were separated by 12% sodium dodecyl sulfate‐polyacrylamide gel electrophoresis and transferred to polyvinylidene fluoride membranes. Membranes were probed with primary antibodies, and then with secondary. Bands were visualized using chemiluminescence and quantified using densitometry.

### 2.5 Enzyme‐Linked Immunosorbent Assay (ELISA) for Cytokine Release

The release of cytokines in cell supernatants was measured using commercially ELISA kits. Briefly, supernatants were collected from treated podocytes and centrifuged to remove debris. Samples were analyzed in duplicate, and cytokine concentrations were determined by comparing absorbance values to a standard curve.

### 2.6 Chromatin Immunoprecipitation (ChIP) Assay

ChIP assay was conducted using the SimpleChIP Plus Enzymatic Chromatin IP Kit (Cell Signaling Technology, USA). Briefly, NMCs were crosslinked. The chromatin was collected and sonicated into fragments ranging from 200 to 700 bp. Then the sonicated chromatin solutions were incubated with antibodies against STAT3 (ab267373; Abcam, UK) or IgG (ab172730; Abcam, UK). Protein A + G agarose beads were then added. The DNA samples were collected and purified. Subsequently, the purified DNA samples were subjected to PCR.

### 2.7 Luciferase Assay

NMCs were plated into a 24‐well plate. Cells were transfected with pCMV‐3×FLAG‐VEZF1. Wild‐type (WT) and mutant (MUT) AIM2 promoter‐driven firefly luciferase reporters cloned into the pGL3‐Basic vector and transfected into NMCs. After 48 h, luciferase activities were analyzed using a luciferase assay system (Promega, USA).

### 2.8 Cell Counting Kit (CCK‐8) Assay

Cell viability was assessed using the CCK‐8 assay. Briefly, podocytes were seeded in 96‐well plates and treated with puromycin as described. After treatment, 20 µl of CCK‐8 solution (5 mg/ml) was added to each well, and cells were incubated for 4 h at 37°C. Optic values were detected using a microplate reader at 450 nm.

### 2.9 Lactate Dehydrogenase Leakage (LDH) Assay

Cytotoxicity was detected using an LDH kit (C0016; Beyotime, China). Podocytes were seeded into a 96‐well plate. After treated with GL and/or PAN, cells were centrifuged. The supernatants were collected and supplemented with LDH regents. After centrifugation, the supernatants were collected and subjected to a microplate reader at the wavelength of 490 nm.

### 2.10 Immunofluorescence and Terminal Deoxynucleotidyl Transferase dUTP Nick‐End Labeling (TUNEL) Assay

For immunofluorescence assay, cells were fixed and permeabilized. Cells were fixed and permeabilized. Cells were blocked with 5% bovine serum albumin (BSA) and incubated with primary antibodies against LC3 puncta. Nuclei were counterstained with 4′,6‐diamidino‐2‐phenylindole (DAPI). Images were captured using an immunofluorescence microscope.

Cell death was determined by an In Situ TUNEL kit (C1086; Beyotime, China). Briefly, cells grown on coverslips were fixed and permeabilized. Cells were blocked with 5% BSA for 1 h and added with TUNEL regents. Nuclei were counterstained with DAPI. Images were captured using an immunofluorescence microscope.

### 2.11 Statistical Analysis

Data were analyzed using SPSS19.0 and presented as mean ± standard deviation. Statistical difference analysis was performed using student *t*‐test and ANOVA followed by Turkey's post hoc test. *p* < 0.05 was considered statistically significant.

## 3 Results

### 3.1 MF Suppresses PM_2.5_‐Induced Inflammation in NMCs

**Figure 1:**
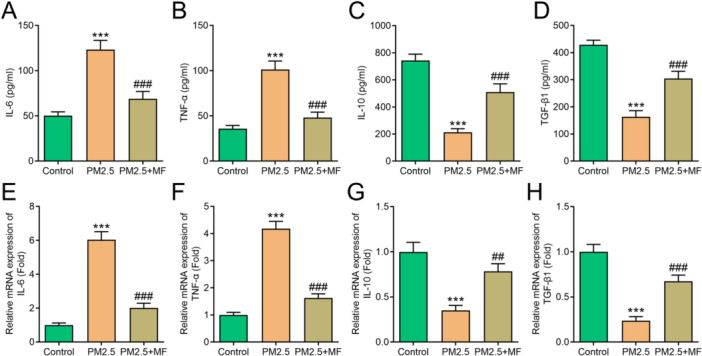
MF suppresses PM2.5‐induced inflammation in NMCs. (A–D) ELISA was conducted to detect cytokine release. (E–G) RT‐qPCR was used to detect mRNA levels. **p < 0.01, ***p < 0.001.

Long‐tern exposure to PM_2.5_ contributes to inflammation and the progression of chronic rhinitis [14]. We found that PM_2.5_ exposure markedly increased the release of pro‐inflammatory cytokines, such as IL‐6 and tumor necrosis factor (TNF)‐α (Figure 1A,B), whereas reduced anti‐inflammatory cytokines IL‐10 and transforming growth factor β (TGF‐β) (Figure 1C,D). However, MF treatment reversed the effects of PM_2.5_ and inhibited the release of IL‐6 and TNF‐α, and increased IL‐10 and TGF‐β (Figure 1A–D). These is paralleled with the results from RT‐qPCR assay. As shown in Figure 1E–H, MF treatment significantly suppressed the mRNA levels of IL‐6 and TNF‐α, whereas increased IL‐10 and TGF‐β IL‐6 and TNF‐α.

### 3.2 MF Suppresses PM_2.5_‐Induced Pyroptosis of NMCs

**Figure 2:**
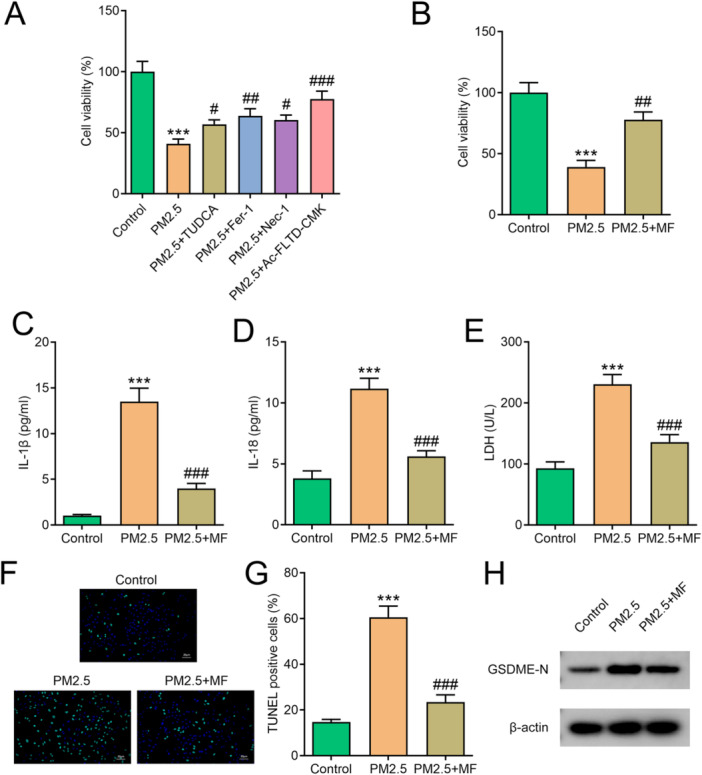
MF suppresses PM_2.5_‐induced pyroptosis of NMCs. (A and B) CCK‐8 was applied for determining cell viability. (C and D) ELISA was conducted to detect cytokine release. (E) LDH assay was conducted for detecting cytotoxicity. (F‐G) TUNEL assay was conducted for detecting cell death. Scale bar: 20 μm. (H) Western blot was used to detect GSDME protein expression. **p < 0.01, ***p < 0.001.

Continuous exposure to PM_2.5_ promotes pyroptosis of cells [15]. To verify whether MF induces PM_2.5_ exposure induces the pyroptosis of NMCs, we analyzed the functions of NMCs after treatment with PM_2.5_ and/or apoptosis inhibitor (TUDCA), ferroptosis inhibitor (Fer‐1), necroptosis inhibitor (Nec‐1), and pyroptosis inhibitor Ac‐FLTD‐CMK. We found that PM_2.5_‐induced inhibition of NMC cell viability was significantly reversed by TUDCA, Fer‐1, Nec‐1, Ac‐FLTD‐CMK (Figure 2A). The effects of Ac‐FLTD‐CMK was more remarkable, suggested that pyroptosis may be the main form of cell death induced by PM_2.5_ exposure. Moreover, we found that MF treatment significantly reversed the effects of PM_2.5_ exposure and restored the cell viability of NMCs (Figure 2B). Moreover, MF treatment remarkably inhibited the effects of PM_2.5_ exposure and suppressed the release of IL‐1β and IL‐18 (Figure 2C,D). MF treatment significantly inhibited the cytotoxicity of NMCs induced by PM_2.5_ exposure (Figure 2E). MF treatment also significantly reversed the effects of PM_2.5_ exposure and suppressed the death of NMCs (Figure 2F‐G). Additionally, MF treatment significantly suppressed the protein expression of N‐terminal of gasdermin E (GSDME‐N) (Figure 2H).

### 3.3 MF Promotes the Activation of Autophagy

**Figure 3:**
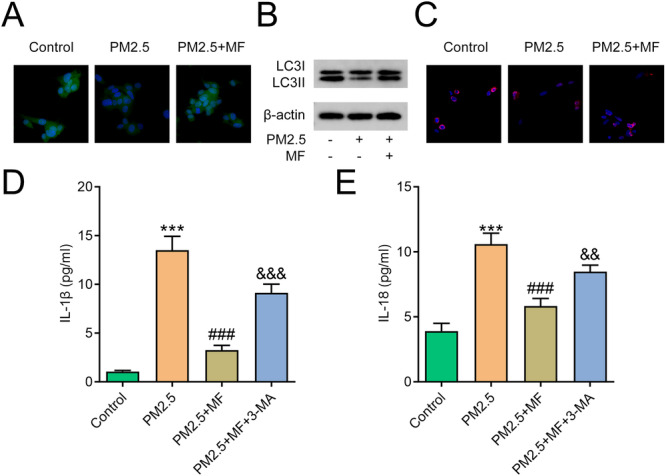
MF promotes the activation of autophagy. (A) Immunofluorescence was conducted to detect lysosome formation. Scale bar: 10 μm. (B) Western blot was used to detect LC3II/I protein expression. (C) Immunofluorescence was conducted to detect the fluorescence intensity of LC3 puncta. Scale bar: 10 μm. (D,E) ELISA was conducted to detect cytokine release. **p < 0.01, ***p < 0.001.

Autophagy‐mediated the formation of lysosome promotes inflammation resolving and cell survival [16]. We found that PM_2.5_ exposure significantly suppressed the formation of lysosome (Figure 3A), which was reversed by MF treatment. Moreover, MF treatment markedly promoted the protein expression of LC3II (Figure 3B) as well as increased the fluorescence intensity of LC3 puncta (Figure 3C). To confirm the roles of autophagy in chronic rhinitis, NMCs were exposed to 3‐MA. 3‐MA markedly reversed the effects of MF and increased the release of IL‐1β and IL‐18 (Figure 3D‐E).

### 3.4 MF Inactivates JAK2/STAT3 Signaling

**Figure 4:**
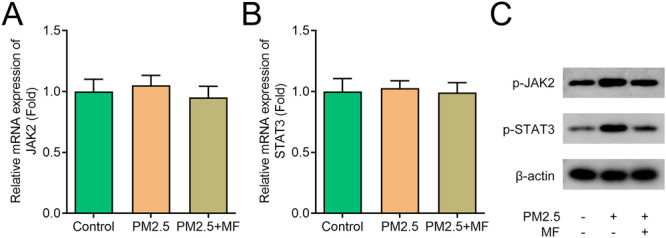
MF inactivates JAK2/STAT3 signaling. (A) RT‐qPCR was used to detect mRNA levels. (B and C) Western blot was used to detect p‐JAK2/STAT3 protein expression. **p < 0.01, ***p < 0.001.

JAK2/STAT3 signaling is the key regulator of inflammation [17]. Therefore, we hypothesized that MF may suppress inflammation via regulating JAK2/STAT3 signaling. We found that the mRNA levels of JAK2 and STAT3 showed no marked alteration after treated with PM_2.5_ and/or MF (Figure 4A,B). However, PM_2.5_ exposure markedly increased the protein expression of p‐JAK2 and p‐STAT3 (Figure 4C), which was reversed by MF treatment.

### 3.5 MF‐Mediated Downregulation of STAT3 Inhibits the Transcription of AIM2

**Figure 5:**
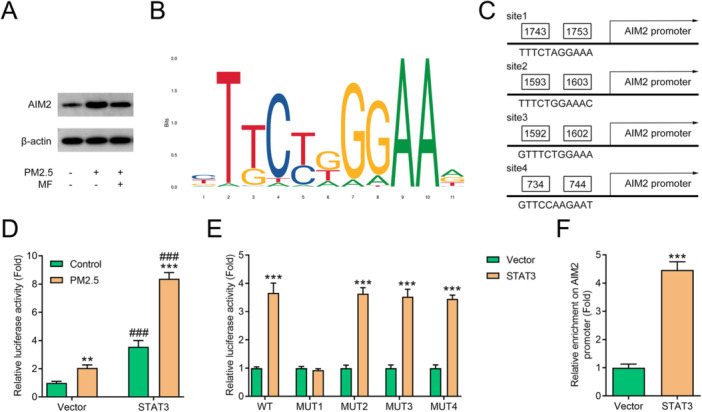
MF‐mediated downregulation of STAT3 inhibits the transcription of AIM2. (A) Western blot was used to detect AIM2 protein expression. (B) JASPAR was used to analyze the binding motif of STAT3. (C) JASPAR was used to predict the binding sites in the promoter of AIM2. (D‐E) AIM2 transcription was detected using luciferase assay. (F) The interaction between STAT3 and AIM2 was confirmed by ChIP assay. **p < 0.01, ***p < 0.001.

The activation of AIM2 inflammasome contributes to the progression of rhinitis [18]. STAT3‐mediated upregulation of AIM2 promotes inflammatory response and the enrichment of pro‐inflammatory immune cells. We found that the protein expression of AIM2 was markedly decreased by MF (Figure 5A), which was reversed by overexpressed STAT3. STAT3, as a transcription factor, regulate gene expression via binding to the promoter of the target. The online database JASPAR (https://jaspar.elixir.no/) predicted the binding motif of STAT3 (Figure 5B). The results from JASPAR showed that there were 4 binding sites in the promoter of AIM2 (Figure 5C). We found that PM_2.5_ exposure markedly enhanced STAT3‐mediated transcription of AIM2 (Figure 5D). Moreover, overexpressed STAT3 significantly increased the luciferase activity in WT, MUT2, MUT3, and MUT4 (Figure 5E), whereas showed no significantly alteration in MUT1. These results suggested that STAT3 may regulate the transcription of AIM2 via binding to site1. ChIP assay showed that significantly increased JAK2/STAT3 signaling co‐occupied the wild‐type binding site1 (Figure 5F), which was enhanced by PM_2.5_ exposure. These results suggested that MF suppresses STAT3‐mediated transcription of AIM2 in PM_2.5_ exposure‐induced chronic rhinitis.

### 3.6 Activation of AIM2 Inflammasome Promotes Inflammatory Response

**Figure 6:**
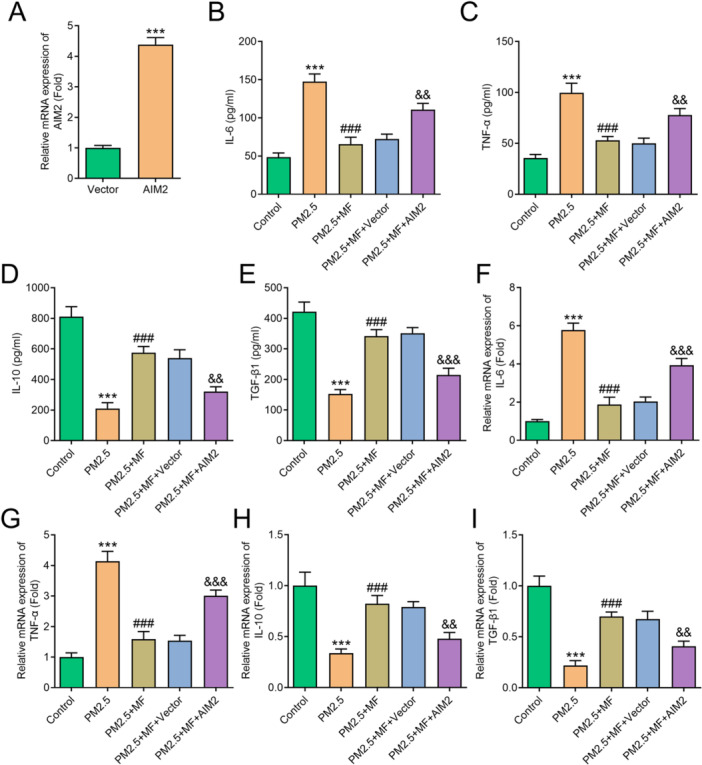
Activation of AIM2 inflammasome promotes inflammatory response. (A) RT‐qPCR was used to detect mRNA levels. (B–E) ELISA was conducted to detect cytokine release. (F–I) RT‐qPCR was used to detect mRNA levels. **p < 0.01, ***p < 0.001.

Recure assay was conducted to confirm the role of AIM2 inflammasome in chronic rhinitis. Figure 6A showed the transcription efficiency of AIM2 overexpression plasmids. We found that overexpressed AIM2 significantly reversed the effects of MF and promoted the release of IL‐6 and TNF‐α (Figure 6B,C), whereas decreased IL‐10 and TGF‐β (Figure 6D,E). Moreover, overexpressed AIM2 significantly increased the mRNA levels of IL‐6 and TNF‐α (Figure 6F,G), whereas decreased IL‐10 and TGF‐β (Figure 6H,I).

### 3.7 AIM2 Mediates Pyroptosis of NMCs

**Figure 7:**
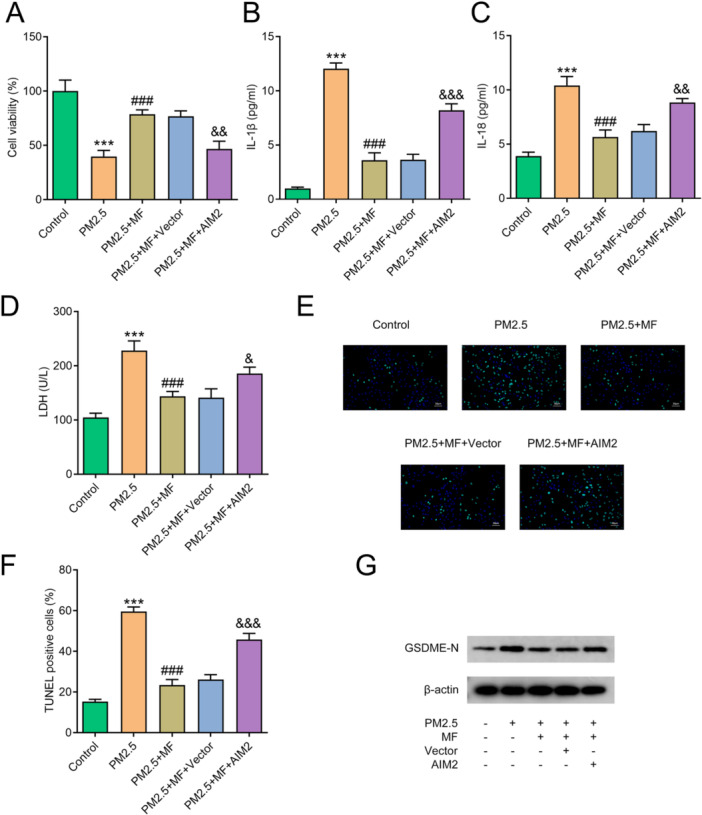
AIM2 mediates pyroptosis of NMCs. (A) CCK‐8 was applied for determining cell viability. (B and C) ELISA was conducted to detect cytokine release. (D) LDH assay was conducted for detecting cytotoxicity. (E‐F) TUNEL assay was conducted for detecting cell death. Scale bar: 20 μm. (G) Western blot was used to detect GSDME protein expression. **p < 0.01, ***p < 0.001.

Overexpressed AIM2 significantly inhibited the cell viability of NMCs (Figure 7A). Overexpressed AIM2 significantly attenuated the effects of MF and increased release of IL‐1β and IL‐18 (Figure 7B,C). Overexpressed AIM2 markedly increased the LDH levels in NMCs (Figure 7D). Moreover, overexpressed AIM2 significantly promoted the pyroptosis of NMCs (Figure 7E‐F). Overexpressed AIM2 significantly increased the protein expression of GSDME‐N (Figure 7G).

## 4 Discussion

Our study provides novel insights into the role of pyroptosis in the therapeutic effects of MF in rhinitis. The induction of pyroptosis by PM_2.5_ exposure may contribute to the resolution of inflammation in nasal mucosa. This mechanism may complement the traditional anti‐inflammatory actions of MF, such as suppression of cytokine production and inhibition of inflammatory cell recruitment [19, 20]. Mechanically, MF inactivated JAK2/STAT3 signaling, which inhibited the transcription of AIM2 as well as pyroptosis of NMCs. The clinical trial results further support the therapeutic efficacy of MF in reducing nasal symptoms in patients with chronic rhinitis.

Increasing studies have reported the protective effects of MF in rhinitis. For instance, MF reduces the total oxidative stress in allergic rhinitis [21]. MF inhibits the expansion of Th2 cells via upregulating anti‐inflammatory IL‐37 [22]. Kariyawasam et al. [23] demonstrate that MF inhibits the progression of chronic rhinitis via inactivating IL‐4/IL‐13 signaling. These findings suggest that MF mainly suppresses rhinitis through blocking pro‐inflammatory mechanisms. In this study, MF suppressed the inflammatory response via inactivating JAK2/STAT3 signaling. The activation of JAK2/STAT3 signaling promotes the enrichment of pro‐inflammatory cytokines and the progression of allergic rhinitis [24]. Ding et al. demonstrate that JAK2/STAT3 signaling mediates the CD4 + T cell differentiation and induces nasal mucosa epithelial barrier dysfunction [25]. Interestingly, targeting JAK2/STAT3 signaling using its specific inhibitor suppresses the accumulation of pro‐inflammatory Th17 cells [26]. In this study, MF‐mediated inactivation of JAK2/STAT3 signaling suppressed inflammatory response and subsequent pyroptosis of NMCs.

AIM2 inflammasome is frequently upregulated during the progression of rhinitis [8]. The activation of AIM2 induces nasal mucosa damages [7]. Our study provides novel insights into the role of AIM2‐mediated pyroptosis in NMCs and its contribution to chronic rhinitis pathogenesis. The increased expression and activation of AIM2 in nasal mucosa cells exposed to PM_2.5_ highlight its role in driving inflammation and tissue remodeling. The involvement of AIM2 in pyroptosis and cytokine release underscores its potential as a therapeutic target for reducing inflammation and improving clinical outcomes in chronic rhinitis.

The findings of this study have several clinical implications. First, pyroptosis induction may be a key factor in the long‐term efficacy of MF, potentially reducing the need for continuous high‐dose corticosteroid therapy. Second, the ability to modulate pyroptosis may offer a new therapeutic strategy for refractory rhinitis cases that do not respond to traditional anti‐inflammatory treatments. Future research should focus on elucidating the upstream signaling pathways involved in pyroptosis induction by MF and exploring the potential benefits of combining it with other pyroptosis‐inhibiting agents.

This study has several limitations. First, all the experiments were performed based on in vitro models. Further studies will be warranted to involve in vivo experiments as well as clinical practice. MF may inhibit inflammatory response in rhinitis through regulating anti‐inflammatory SOCS3. Second, apart from regulating STAT3 signaling, whether MF alleviates PM_2.5_‐induced chronic rhinitis via regulating anti‐inflammatory signaling. This needs further studies.

## 5 Conclusion

MF inhibits pyroptosis in nasal mucosa cells via JAK2/STAT3/AIM2 signaling, contributing to its therapeutic efficacy in rhinitis management. This study highlights the importance of targeting inflammation‐related pyroptosis in resolving inflammation and improving clinical outcomes in patients with chronic rhinitis. Further investigations into the mechanisms of pyroptosis inhibition by MF may lead to the development of more effective and targeted therapies for rhinitis and related inflammatory conditions.

## Author Contributions

B.S. designed the experiments. Z.Q.L and G.L. performed the experiments. B.S., Z.Q.L., and G.L. collected and analyzed the data. Z.Q.L. and G.L. drafted manuscript. All authors read and approved the final manuscript.

## Funding

The authors have nothing to report.

## Ethics Statement

All patients were informed and signed informed consent voluntarily. This study was approved by the ethics committee of the Hubei Maternal and Child Health Hospital and complied with the guidelines outlined in the declaration of Helsinki were followed. The written consent was received from all participants.

## Consent

The authors have nothing to report.

## Conflicts of Interest

The authors declare no conflicts of interest.

## Data Availability

The data that support the findings of this study are available from the corresponding author upon reasonable request.
